# Creative purpose

**DOI:** 10.1017/S2045796020000190

**Published:** 2020-03-12

**Authors:** Marc Steene

**Affiliations:** Outside In, Chichester, West Sussex

**Keywords:** Art brut, contemporary art, outsider art, raw art, stressful life events

Outsider art is often created for different purposes from the majority of conventional arts practice. The work is often deeply connected with the artist's life, a means to express inner worlds and emotions. It often does not worry itself about audiences, contemporary art contexts or commodity. This art is not necessarily for sale, or even to be shared or seen. It is a tool for healing, sometimes to make sense of the artist's life and sometimes to process and understand the unimaginable. There is a charge and immediacy to this work, it strikes one with its bravery and honesty, it poses a challenge to curators and viewers alike with its individuality. We are made uncomfortable by this art that does not reference anything else other than the individual and their experience.

Two artists who I feel epitomise their use of creativity to articulate the harrowing and unique experiences they faced are Wilhelm Werner (1898–1940) and Drew Fox (b. 1965). I included both artists in *The Outside and the Inside* (Steene, 2019) an exhibition I curated at The Lightbox in Woking in 2019 which explored the purposes and motivations behind non-academic and private art.

**Figure:**
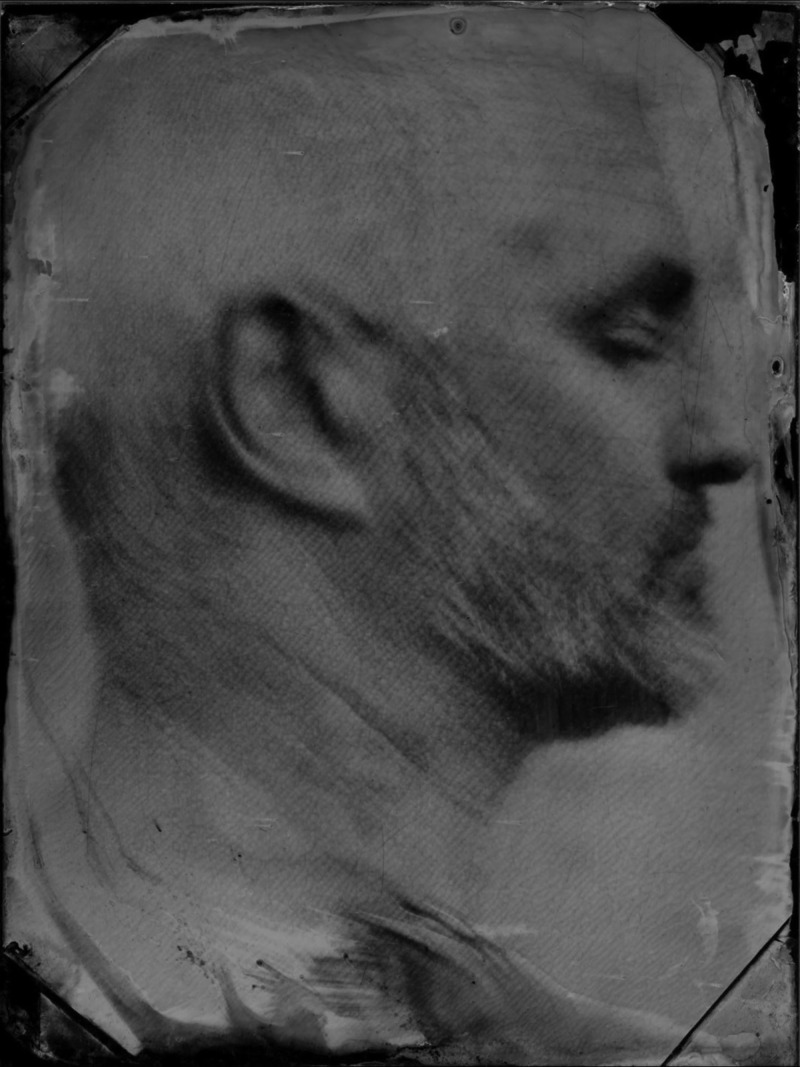
Drew Fox (b. 1965) When water turns to steel (the alchemy of death), not dated. Wet Plate Collodion Photograph. Copyright the artist.

Werner's art was recently discovered and acquired by the Prinzhorn Collection (Werner *et al*., 2014; Connolly, 2020). His work consists solely of a book of drawings whose personal testimony shines a powerful light on what is both a shocking personal experience and a unique documentation of a person with learning disabilities from the Nazi Holocaust. Werner was diagnosed with ‘idiocy’ and compulsory sterilised by the Nazis sometime between 1934 and 1938 as part of their ‘NS-Rassenhygiene’ race cleansing programme and later gassed as part of their euthanasia programme on 6th October 1940 at the Pirna-Sonnenschein death camp.

In all there are 44 drawings by Werner, of which 30 are numbered in sequence. There is a theatricality to the drawings, it is as if they are illustrating some bizarre Weimar period marionette performance. On the inside of the book cover Werner describes himself as ‘…orator of the people and theatre director Wilhelm Werner, Werneck Asylum’. The story that these mannequins tell is harrowing, Werner directs a shocking series of tableaus, all drawn in a highly controlled way, exploring his sterilisation. His characters are seeming puppets, victims lacking autonomy and under the control of cigarette smoking Nazi nurses.

Werner created his drawings to try and make senses of the terrifying situation he found himself in, we can assume that he had no sense that these would ever be seen or shared. These drawings are remarkable in their control and in the artist's ability to craft his tragedy as a piece of theatre. We should not overlook the intention in Werner's work, this is a deliberate act of creation, an act of defiance and a statement of personal suffering drawn with great control and bravery.

**Figure:**
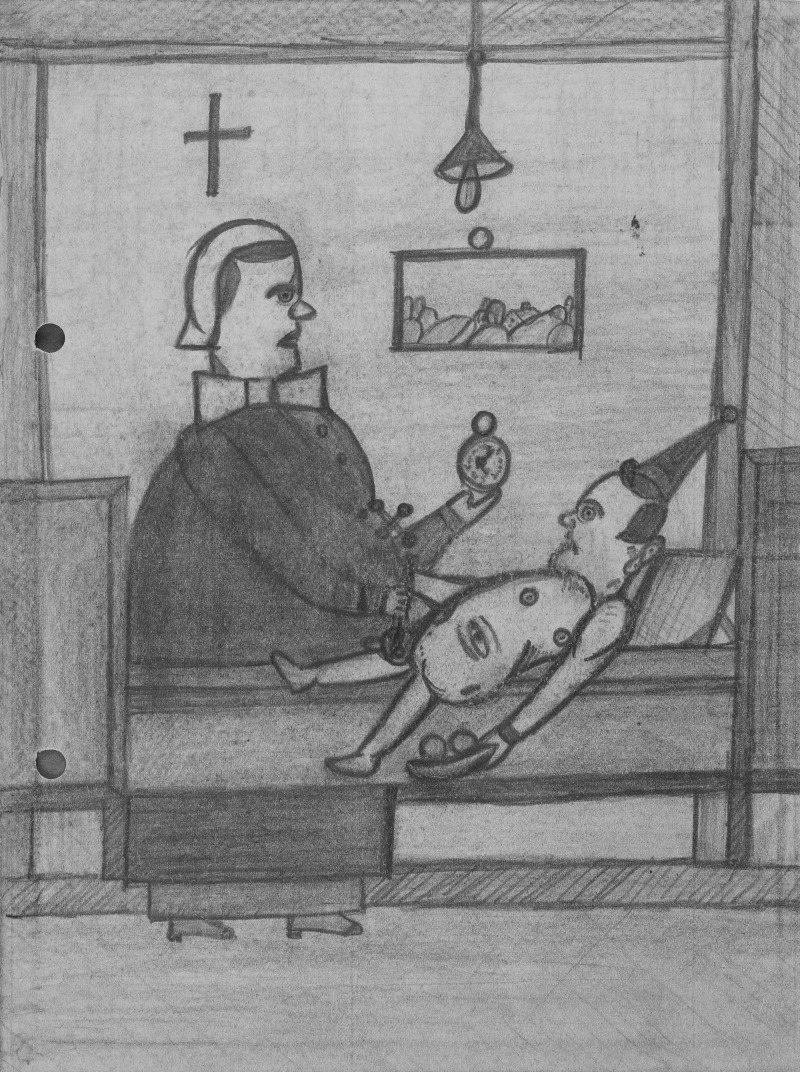
Wilhelm Werner (1898–1940), Sterelation Drawings, ca. 1934–1938. Pencil on paper. Prinzhorn Collection Heidelberg University Hospital. Copyright Prinzhorn Collection.

Fox's powerful photographic self-portraits deal with his mortality hauntingly conveyed in wet plate collodion photographs and film (Fox, 2019). Fox had a near death experience when he was pronounced clinically dead and resuscitated twice. This had a profound impact on him and led to a body of work in which he expressed and explored the feelings and sensations he experienced whilst between life and death.

Fox researched early photographic techniques and built his own cameras out of biscuit tins and gaffer tape, not dissimilar to the well-known outsider photographer Miroslav Tichý (Sanguinetti, 2011). Fox's cameras are made with a special purpose, to pierce the veil of life and reveal the landscape he experienced on the other side. Especially powerful is a series of four self-portraits, *When water turns to steel (the alchemy of death)*. The images represent the four stages of his death, each image lighter than the last, fading as Fox felt he lost his humanness. Fox said of these photographs ‘I think these images were the most upsetting to make as they describe my death and how it felt to die’ (Steene, 2019).

Neither Werner nor Fox produced their work for public consumption, their creativity a tool for them to deal with their traumatic experiences. Their work is difficult to look at and the stories behind them difficult to hear, but this is art nonetheless and even more resonant and powerful for its cathartic and personal content. It might seem voyeuristic or almost disrespectful to show this work and we might ask what will audiences gain from engaging with it? I would argue that seeing this work is vital reminding us that art is not always about the things we think it should be, that understanding that creativity is a tool we all can access to help us understand and express our lives is vital for the health and wellbeing of all. Driven to create, art can be the ultimate defiance and means to makes sense of the lives we live.

## About the author

Marc Steene is the director and founder of the award winning charity Outside In and the vice president of the European Outsider Art Association. Formerly the executive director of Pallant House Gallery he is an experienced figure in the gallery and museum sector, a strategic thinker, partnership broker and leader in his field.

As well as his ground breaking work in community engagement he has been responsible for the programming and curating of acclaimed exhibitions of outsider, modern, historic, international and contemporary art including *Nick Blinko: The Visions of Pope Adrian 37^th^*, Pallant House Gallery (2011); *Friedrich Nagler: Wunderkammer*, Pallant House Gallery (2016) and *The Outside and the Inside,* Lightbox Gallery (2019).

Carole Tansella, *Section Editor*

## References

[ref1] Connolly K (2020) ‘Miraculous survival’: asylum patient whose art documented his sterilisation by the Nazis. The Guardian. Available at https://www.theguardian.com/world/2020/jan/06/miraculous-survival-asylum-patient-whose-art-documented-his-sterilisation-by-the-nazis. Published online: 6 January 2020.

[ref2] Fox D (2019) To Death and Back. Available at https://www.youtube.com/watch?v=YE7Zt982fUs&feature=emb_title. Published online: 23 January 2020.

[ref3] Sanguinetti G (2011) Miroslav Tichy. Les Formes du Vrai/Forms of Truth. Prague: Kant.

[ref4] Steene M (2019) Outside In and the Ingram Inside Out Collections: The Outside and The Inside, Exhibition, The Lightbox Gallery: Woking.

[ref5] Werner W, Röske T and Rotzoll M (2014) Wilhelm Werner: Sterelationszeinchnungen. Heidelberg: Wunderhorn Verlag.

